# Integrin-Src-YAP1 signaling mediates the melanoma acquired resistance to MAPK and PI3K/mTOR dual targeted therapy

**DOI:** 10.1186/s43556-020-00013-0

**Published:** 2020-11-10

**Authors:** Chune Yu, Min Zhang, Jinen Song, Xiaobo Zheng, Guangchao Xu, Yu Bao, Jiang Lan, Dan Luo, Jianping Hu, Jingyi Jessica Li, Hubing Shi

**Affiliations:** 1 Laboratory of Tumor Targeted and Immune Therapy, Clinical Research Center for Breast, State Key Laboratory of Biotherapy, West China Hospital, Sichuan University, No. 17, 3rd Section, Renmin South Road, Chengdu, 610041 Sichuan China; 2 Department of Plastic Surgery, Affiliated Hospital of Zunyi Medical University, Zunyi, 563000 Guizhou China; 3 Department of Immunology, School of Basic Medical Sciences, Chengdu medical College, Chengdu, 610500 Sichuan China; 4 College of Pharmacy and Biological Engineering, Sichuan Industrial Institute of Antibiotics, Key Laboratory of Coarse Cereal Processing, Ministry of Agriculture and Rural Affairs, Chengdu University, Chengdu, 610106 China; 5 Department of Statistics, University of California, Los Angeles, CA 90095-1554 USA

**Keywords:** Melanoma, Targeted therapy, Resistance, MAPK, PI3K/mTOR, Integrin-Src-YAP1 axis

## Abstract

Activation of PI3K/AKT pathway is one of the most recurrent resistant mechanisms for BRAF-targeted therapy, and the combination of MAPK and PI3K/AKT inhibitors becomes one of the most promising regimens for BRAF-targeted relapsed melanoma patients. Although the potent drug efficacy was observed in preclinical experiments and early clinical trials, the dual-drug resistance is inevitable observed. In this study, we systematically explored the mechanisms of dual-drug resistance to MAPKi and PI3K/mTORi in melanoma. With transcriptomic dissection of dual-drug resistant models, we identified that the drug tolerance was mediated by ECM-integrins α3β1 and α11β1 signaling. Upon binding ECM, the integrins activated downstream kinase Src rather than FAK, WNT, or TGFβ. Knockdown of integrins α3, α11, and β1 significantly inhibited the proliferation of dual-drug resistant sublines while with trivial effects on parental cells. Although Src inhibition suppressed the phosphorylation of AKT, c-JUN, and p38, none of inhibitors targeting these kinases reversed the dual-drug resistance in model cells. Notably, Src inhibitor promoted the phosphorylations of LATS1 and YAP1, subsequently, re-localized YAP1 from nucleus to cytosol facilitating further degradation. Both small molecule inhibitors and shRNAs targeting YAP1 or Src overcame the MAPKi and PI3K/mTORi dual-drug resistance. In conclusion, our data not only illuminated an integrin-Src-YAP1 pathway mediated MAPKi and PI3K/mTORi dual-drug resistant mechanism but also provided a potential combinatorial regimen for the drug-relapsed melanoma patients.

## Introduction

As one of the most common malignant cancer worldwide, metastatic melanoma contributes to the highest mortality among all types of skin cancer [1]. About 60% cutaneous melanomas harbor a BRAF^V600E^ mutation, which sustainedly activated BRAF/MEK/ERK (MAPK) signal pathway for malignant transformation and carcinogenesis [2]. Targeting BRAF^V600E^ with small molecules in metastatic melanoma has been shown promising outcomes in preclinical experiments and clinical trials [3, 4]. Small molecules blockading BRAF^V600E^ not only shrink tumor mess but also relief comprehensive symptoms in clinics [5]. To date, three compounds have been approved by FDA as either monotherapy or combinational therapy for the treatment of unresectable metastatic melanoma [6]. After prompt initial response, tumor relapse was observed universally in 9 to 10 months, which was driven by the development of acquired or adaptive resistance [7].

There are two main aspects of the molecular mechanisms for drug resistance of targeted BRAF^V600E^ therapy [8]. One is reactivation of MAPK pathway [9–14], which could be inhibited by co-targeted with BRAF and MEK inhibitors (BRAFi and MEKi). Combination targeted therapy with BRAFi and MEKi could delay the tumor relapse in clinics [15, 16], but there are still other alternative pathways activated to drive drug resistance. The other is the activation of alternative survival networks, the majority of which involved in PI3K/AKT pathway, such as up-regulation of RTKs [11, 17, 18], loss of PTEN [19, 20], PI3K mutation [9, 21], and AKT1/3 mutation [22]. In addition, resistance to MAPK inhibitors mediated by phenotypic plasticity were reported in recent years [23]. The dual activation of MAPK and PI3K/mTOR pathways is likely to be the reason for single pathway inhibition resistance [24]. Several preclinical studies have shown that combination therapy with MAPKi and PI3K/mTORi could broaden antitumoral spectrum and inhibit tumor cells growth, consequently delayed tumor relapse [25, 26]. Villanueva et al. also showed that co-targeting MEK and IGF-1R/PI3K could re-sensitize resistant melanoma to BRAF inhibitor [17]. Encouraged by these favorable results from preclinical models, several clinical trials have been initiated to evaluate the safety and effects of combination therapy with MAPKi and PI3K/mTORi in melanoma patients. For instance, a phase I clinical trial have shown that co-targeted therapy via MEKi and PI3K/mTORi yielded a better response compared with single drug alone [27]. Although combination targeted therapies show prosperous prospects for the treatment of metastatic melanoma, the development of drug resistance is still inevitable with times. Further investigation is urgently needed in advance to elucidate the molecular mechanisms of dual-drug resistance.

In this study, we aim to investigate the compensatory pathway of dual-drug resistance and seek for a potential novel combinatorial regimen for overcoming tumor relapse. To this end, we firstly established dual-drug resistant cell models and characterized the transcriptomic profiles of isogenic pairs including parental cells and resistant cells. We found that ECM receptor signaling pathways were intensively enriched during the progress of dual resistance development. Integrins α3, α11, and β1, the receptor of ECM signaling pathway, were upregulated on the membrane of dual-drug resistant cells. Then, functional assay indicated that integrins α3, α11, and β1 potentially mediate resistance to combination of MAPKi and PI3K/mTORi. Notably, the investigation of molecular mechanism underlying this acquired dual-drug resistance highlights an integrins-Src-YAP1 axis. Targeting this pathway with small molecules or shRNA re-sensitizes resistant cell lines, which provides a promising combinatorial regimen for MAPKi and PI3K/mTORi dual-drug resistant melanoma patients.

## Results

### Chronic exposure to MAPKi and PI3K/mTORi leads to dual-acquired drug resistance

**Fig. 1:**
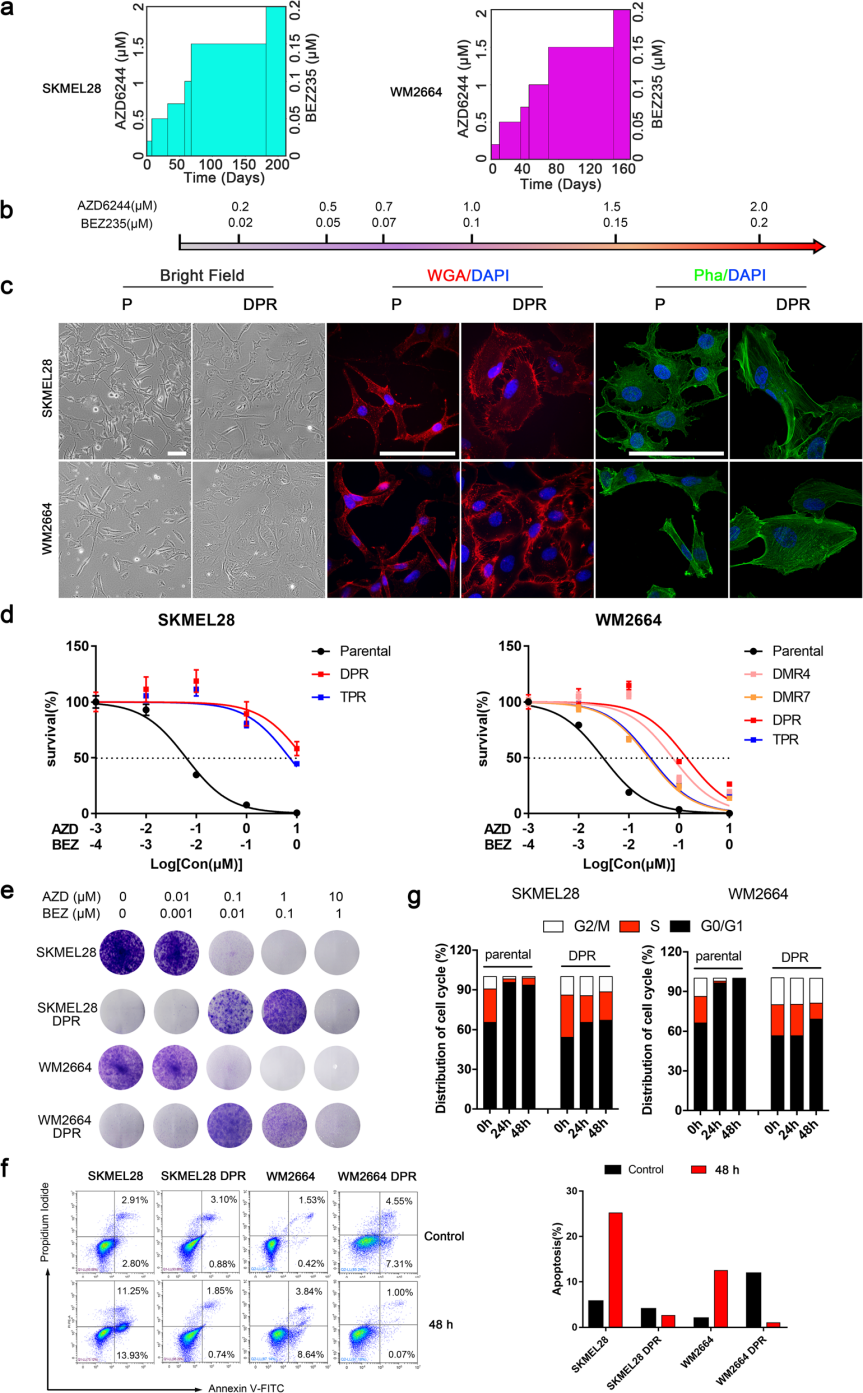
Chronic MAPK and PI3K dual-inhibition lead to acquired drug resistance. **a** Relative drugs exposure time to achieve resistance to MEKi+PI3K/mTORi in WM2664 and SKMEL28. **b** Drug naïve cells were chronically treated with increasing concentration of MEK inhibitor AZD6244 and PI3K/mTOR inhibitor BEZ235. **c** Phase-contrast images showing morphological changes in WM2664 and SKMEL28 parental and resistant cell lines (left), immunofluorescence staining for visualizing cell boundaries by fluorescence microscope (middle), immunofluorescence staining for cytoskeleton by confocal (right), (scale bar = 100 μm). **d** Survival curves of parental and dual-drug resistant cell lines titrated with the AZD6244 and BEZ235 or combine for 72 h. Results are shown relative to DMSO-treated controls (mean ± SEM, *n* = 5; dashed line, 50% inhibition). **e** Long-term colony formation assays of melanoma isogenic pairs. Parental and resistant clones were treated with indicated concentration of AZD6244 and BEZ235 for 12–14 days and then stained with 0.05% crystal violet to assay viability. The image is representative of three biological replicates. **f** Parental and DPR sublines were treated with DMSO or AZD6244 (1 μM) + BEZ235 (0.1 μM) for 48 h. Cells were collected and apoptosis was assessed via Annexin V-FITC staining. Quantification of the percentage of apoptosis cells (right). **g** Cell cycle analysis were assessed propidium iodide staining in parental and dual-drug resistant sublines treated with DMSO or AZD6244 (1 μM) plus BEZ235 (0.1 μM) for 24 or 48 h

To investigate the molecular mechanisms of dual-acquired resistance to MEKi and PI3K/mTORi in melanoma, we established MEKi and PI3K/mTORi dual-drug resistant models by two approaches, polyclonal screening and monoclonal screening (Supplementary Fig 1a). Two MAPKi sensitive cell lines with BRAF^V600E^ mutation, WM2664 and SKMEL28, were selected as parental cell models to generate resistant sublines (Supplementary Fig. 1b). Dual-monoclonal resistance (DMR) sublines (WM2664 DMR4, DMR7, DMR9, DMR18) were obtained by cloning pickup from cell population survived from a chronic fixing high dose drug treatment, while dual-polyclonal resistance (DPR) (WM2664 DPR, SKMEL28 DPR) were established by exposing to incremental concentrations of inhibitors up to AZD6244 2 μM + BEZ235 0.2 μM (Fig. 1a, b). In order to completely inhibit MAPK pathway reactivity, we established triple drugs resistant (TPR) models based on top of DPR. All these resistant sublines were maintained with regular DMEM median supplied with AZD6244 1 μM and BEZ235 0.1 μM. In line with previous report [11], a typical MAPK inhibition related cell shape deform was observed gradually during the development of DPRs. When compared to their parental cell lines, both WM2664 and SKMEL28 resistant sublines tend to be flatter and spread more extensively by visualizing cell boundaries (Fig. 1c and Supplementary Fig. 1c). Moreover, different DMRs displayed different cellular morphology. Detail phalloidin staining showed that the actin fiber strengthened accompanied with the morphology change, implying a cytoskeleton related pathway may be activated during the development of drug resistance (Fig. 1c).

The resistance of developed DMR and DPR sublines was validated by three-day MTT assay. As expected, DPR, DMR, and TPR cell lines showed significant resistance to dual inhibitors treatment, and the IC50 increased by 10–100 folds compared to their parental lines (Fig. 1d), respectively. The resistance capabilities were also validated by long-term clone formation assays (Fig. 1e). In addition to cell growth suppression, apoptosis and cell cycle arrest induced by AZD6244 and BEZ235 were significantly reduced in DPRs compared to their parental cells (Fig. 1f, g). In short, the isogenic resistant cell lines well tolerated the blockade of MAPK and PI3K pathways. Based on our in vitro data, we then wanted to investigate the effect of treatment with dual drugs in mice model. To this end, we inoculated C57 with SMM102 [28], a BRAFi sensitive mouse melanoma cell line (Supplementary Fig. 1b). As expected, tumor growth was significantly inhibited with the combination treatment of AZD6244 and BEZ235 without extra toxicity, followed by a regrowth after 2 weeks (Supplementary Fig. 1d-f). Collectively, these results indicated that acquired resistance is inevitable.

### Drug tolerance of melanoma cells to MAPK/PI3K dual-inhibitor was supported by compensatory pathways

**Fig. 2:**
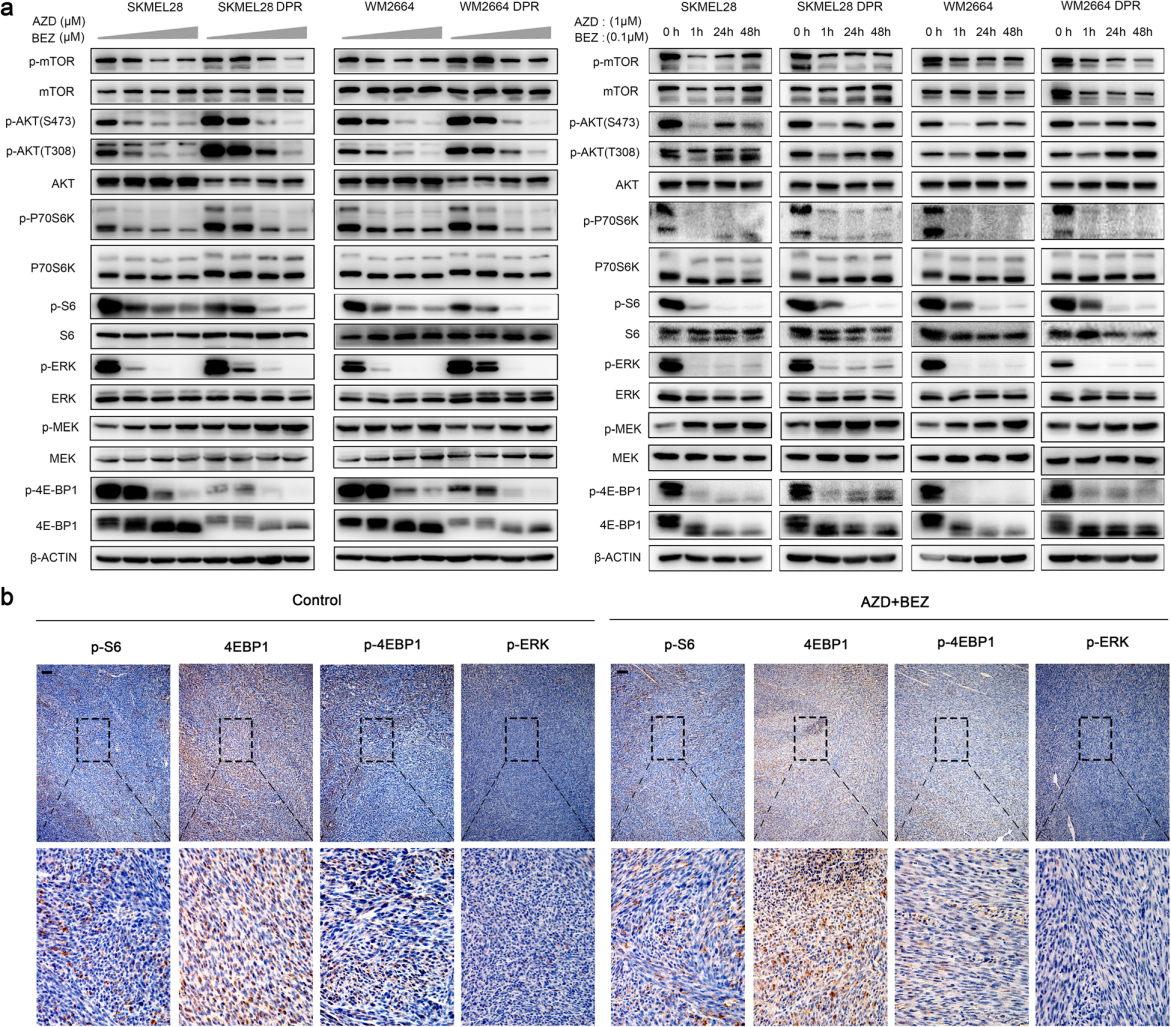
Compensatory signaling supported the survival of melanoma cells with MAPK and PI3K dual inhibition. **a** Dose-dependent suppression of MAPK and PI3K pathways by AZD6244 (0, 0.1, 1, 10 μM) combined with BEZ235 (0, 0.01, 0.1, 1 μM) in WM2664 and SKMEL28 parental and DPRs (left). Phosphorylation levels of these proteins in parental or DPRs treated with AZD6244 (1 μM) and BEZ235 (0.1 μM) for indicated durations (h) (right). **b** IHC analysis of indicated proteins in tumors grown with or without treatment of AZD6244 and BEZ235 (scale bar = 100 μm). Image is representative of five independent experiments

To understand dual-drug resistance mechanisms, we characterized the activation status of kinases or effectors within the MAPK and PI3K/mTOR pathways, including MEK1/2, ERK1/2, AKT, mTOR, p70S6K, S6, 4E-BP1, with isogenic pairs (parental versus dual-drug resistant sublines) in the presence or absence of AZD6244 and BEZ235 by western blot. The results showed that both parental and dual-drug resistant sublines responded to the treatment of MEKi and PI3K/mTORi in a dose-dependent manner. DPRs displayed higher baseline phosphorylation levels of AKT, ERK1/2, and p70S6K (Fig. 2a and Supplementary Fig. 2a). Interestingly, phosphorylation and total level of 4E-BP1 was diminished in resistant cells. The phosphorylation levels of AKT, ERK1/2, and p70S6K in WM2664 DPR and SKMEL28 DPR increased by 10 folds compared to their isogenic parental lines. However, the phosphorylation of S6 and 4E-BP1 showed similar patterns between sensitive and resistant sublines, suggesting that dual-resistance in our model was not render by reactivation of MAPK and PI3K pathways. Nevertheless, there is another potential possibility that the resistance is supported by a delayed compensation feedback [26]. To exclude this point, we profiled the phosphorylation of same kinases and effectors as above in a time course experiment (Fig. 2a and Supplementary Fig. 2b). Sustained inhibitions of p-ERK1/2 p-S6, and p-4E-BP1 were observed up to 48 h, while p-MEK was increased as lacking the inhibition of feedback loop between ERK and RAF when treated with AZD6244 [29]. In addition, although the p-AKT compensations were observed, the suppression of downstream of PI3K pathway effectors, such as p-p70S6K, p-S6, and p-4E-BP1, remained in both parental and resistant cells. Similar to in vitro results, immunohistochemistry (IHC) revealed that p-ERK and p-S6 remained suppressed in dual-drug resistant tumors grown with the treatment of MAPKi and PI3K/mTORi (Fig. 2b). Thus, an alternative redundant pathway independent of canonical PI3K or MAPK cascades might potentially contribute to MAPK and PI3K dual resistance in our models.

### Transcriptome dissection indicated that ECM receptor pathway was involved in MAPK/PI3K dual-inhibitor resistance

**Fig. 3:**
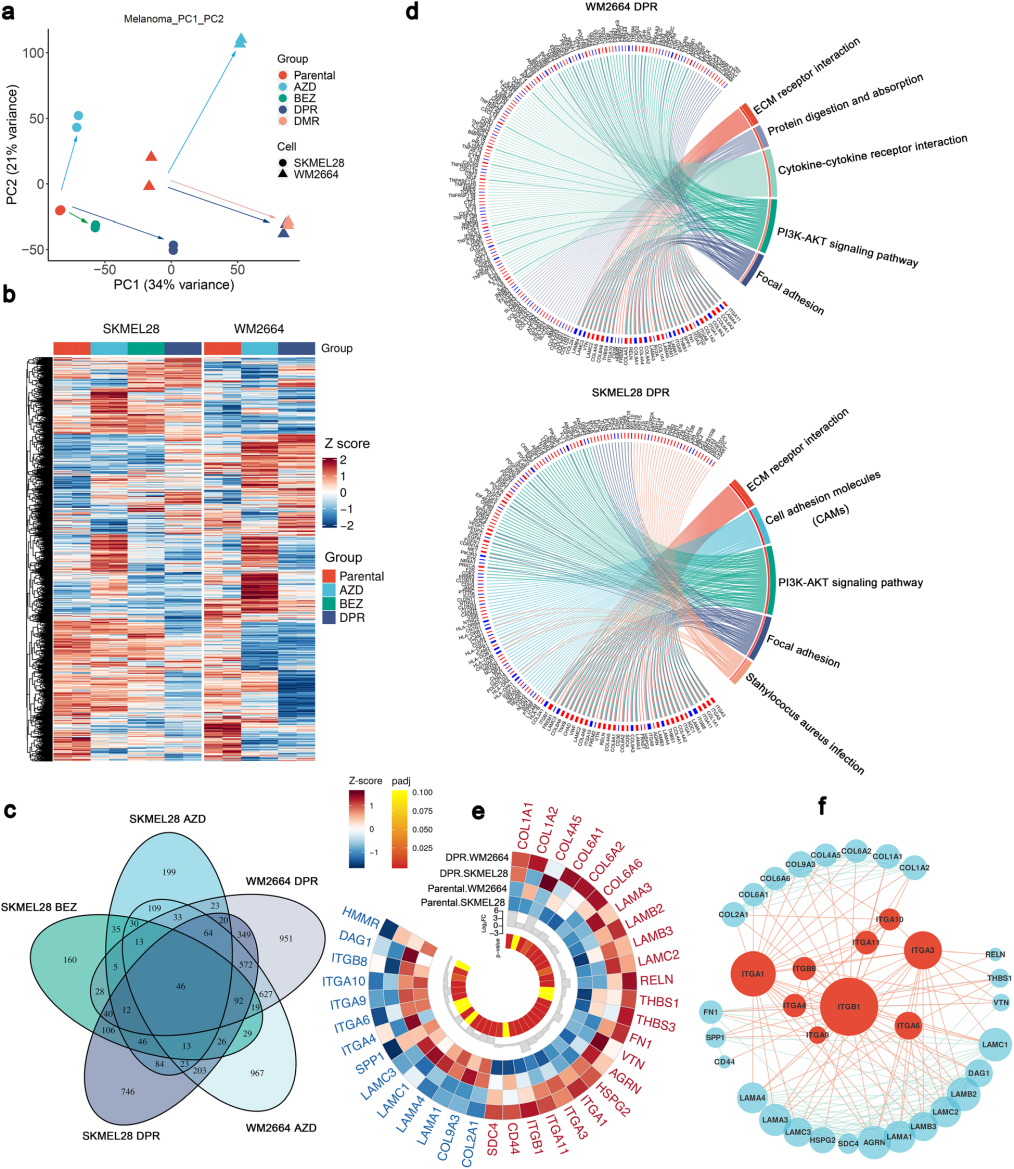
Transcriptome profiling suggested that ECM receptor pathways were enrichment in resistant cell lines. **a** PCA analysis of RNA-seq profiles in WM2664 and SKMEL28 parental, DPRs, and single drug resistance (SDR). Each dot represents one sample. **b** Heat map of all expressed genes in SKMEL28 and WM2664 DPR, SDR, and parental cell lines. Gene-expression variant was calculated by Z-score. **c** Venn diagram of differential expression genes (DEGs) in all resistant cell lines. **d** KEGG enrichment analysis of DEGs in DPR resistant cell lines (*p* < 0.05, top 5). **e** Expression profile of DEGs in the ECM pathway of WM2664 DPR or SKMEL28 DPR, colors of outer circles present z-score of gene expression, and colors of inter circle means *p*-value of Wald statistical test between DPR resistance and Parental cell groups. **f** STRING analysis identified the interaction of differential proteins in the ECM pathway; the size of points indicates the node degree of genes

To investigate the compensatory pathways, we profiled transcriptomes of parental and resistant sublines. Principal component analysis (PCA) showed that isogenic pairs SKMEL28 and WM2664 were discriminated by the primary parameter PC1 (Fig. 3a). Significant difference of gene expression signatures among parental cells, MAPKi or PI3K/mTORi single drug resistant sublines (SDR) (Supplementary Fig. 2c, d), and MAPK/PI3K dual resistant sublines were observed, implying an alternative pathway was activated in the latter. Interestingly, the line of vector from parental to resistance (either single or dual resistance) between these two isogenic pairs (SKMEL28 and WM2664) were almost parallel in the PCA plot (Fig. 3a and Supplementary Fig. 3a), suggesting the two isogenic pairs shared similar acquired resistant mechanisms. This speculation was confirmed by the heatmap of all genes expressed by SKMEL28 and WM2664 parental, SDR and DPR sublines (Fig. 3b). SKMEL28 and WM2664 shared similar pattern with their own isogenic sublines (DPR, SDR) respectively, while the gene-expressed pattern between these two isogenic lines (SKMEL28 vs. WM2664) were significantly different (Fig. 3c).

In order to understand the biological processes of single or dual resistance acquirement, we performed Gene Ontology (GO) enrichment analysis with differential expressed genes (Supplementary Fig. 3b, c). The results indicated that the signatures of extracellular matrix (ECM) organization and extracellular structure organization have been significantly enriched, suggesting that the development of drug resistance is accompanied with extracellular matrix remodeling. Previous study showed that ECM not only played a pivotal role in the regulation of cellular adhesion, morphology, and motility [30], but also mediated drug resistance [31, 32]. That explained why the cell morphology of dual-drug resistant cell lines changed in our model (Fig. 1c and Supplementary Fig. 1c). To confirm this conclusion, we performed an alternative approach independently, Kyoto Encyclopedia of Genes and Genomes (KEGG) pathway enrichment analysis (Fig. 3d). In line with GO analysis, ECM-receptor, cell adhesion molecules (CAMs), and focal adhesion pathways were significantly enriched, indicating that ECM signaling pathway may be the primary pathway conferring the dual-drug resistance.

### ECM-integrin axis drives MAPK/PI3K dual-inhibitor resistance

**Fig. 4:**
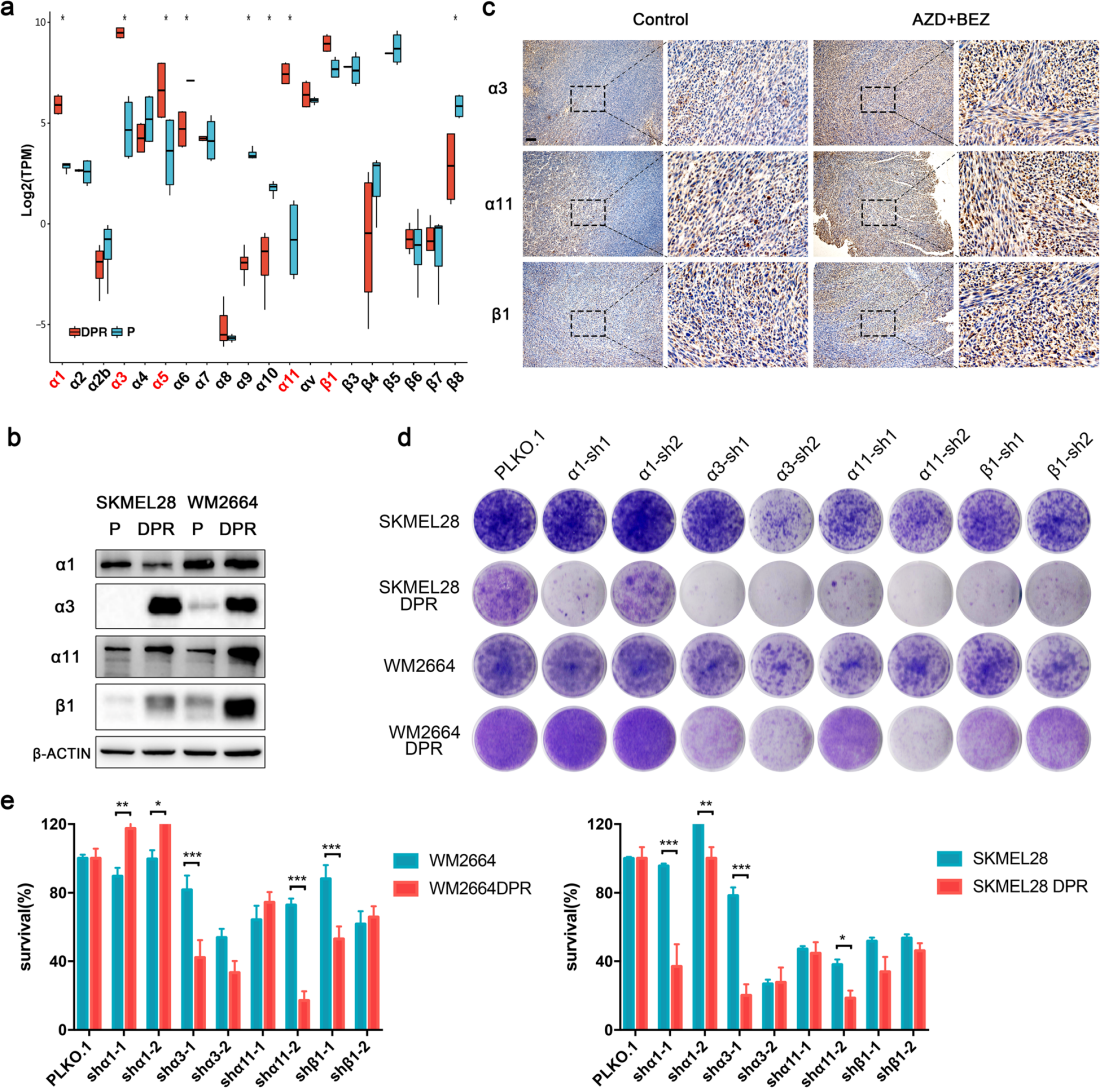
Transcriptome profiling suggested that ECM receptor pathways were enrichment in resistant cell lines. **a** mRNA expression of integrins in SKMEL28 and WM2664 parental and DPRs. Significance was determined by Wilcoxon signed-rank test, ∗*p <* 0.05. **b** Western blot showing protein levels of integrins α1, α3, α11 and β1 in WM2664 and SKMEL28 parental and DPRs. **c** Representative images of IHC analysis with integrin α3, α11 and β1 in tumors grown with or without dual inhibitors (scale bar = 100 μm). Image is representative of five independent experiments. **d** Clonogenic assays of parental and DPRs engineered control (vector) or integrins-targeting shRNAs. Results are shown for one representative of three independent experiments. **e** Quantification of (**d**), ∗*p <* 0.05, ∗∗*p <* 0.01, or ∗∗∗*p <* 0.001

To identify the driver genes that regulated dual-drug resistance, we subsequently interrogated the differential expressed genes in ECM signaling pathways between parental and resistant isogenic pairs. Since SKMEL28 P/DPR and WM2664 P/DPR shared similar pattern during the development of acquired drug resistance, the upregulation of common driver genes should be identified in both two resistant sublines. Total 37 differential expressed genes (*p* < 0.05), both upregulated and downregulated, were identified by Venn diagram (Supplementary Fig. 4a) and visualized by circle heatmap (Fig. 3e). The interaction between the differential expressed genes was analyzed by STRING. The results showed integrins, as the pivotal receptor of ECM signaling pathway, were predominantly upregulated in the resistant sublines (Fig. 3f). Of these integrins, α1, α3, α11 and β1 were significantly upregulated in DPRs (Fig. 4a), but α5 was only upregulated in SKMEL28 DPR compared to their own parental lines. Consistently, western blot results indicated that the protein levels of integrin α3, α11 and β1 were upregulated significantly in DPRs (Fig. 4b). Interestingly, integrin α1 was downregulated in DPRs, suggesting its accelerated protein degradation or inefficient mRNA translation in DPRs. To confirm these observations, we probed these integrins with untreated tumors and tumors treated with MAPKi and PI3K/mTORi by IHC (Fig. 4c and Supplementary Fig. 1f). The results showed that protein levels of these integrins were increased in the drug treated melanomas.

To validate the drug resistance function of integrins, we evaluated the long-term proliferation of parental and DPR sublines with indicated integrins knockdown. The knockdown efficiencies were determined by western blot and real-time q-PCR (Supplementary Fig. 4b-e). The results showed that knockdown of integrin α3, α11 and β1 re-sensitized DPRs to dual inhibitors, while had little effect on parental cells (Fig. 4d, e), indicating that integrin α3β1 and α11β1 play diver roles in dual-inhibitor resistance.

### Integrin-Src axis plays a pivotal role in MAPK/PI3K dual-inhibitor resistance

**Fig. 5:**
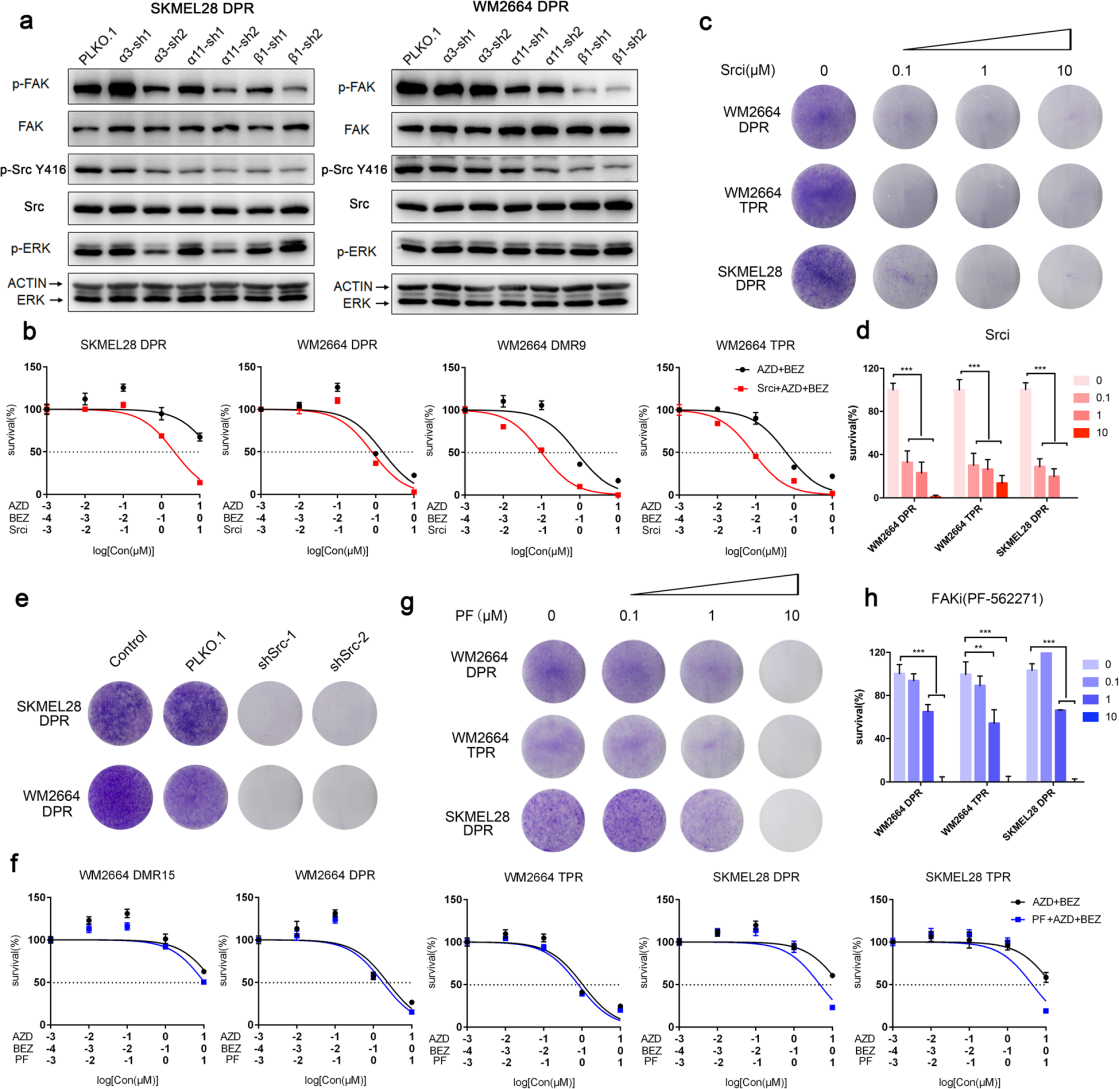
The inhibition of Src suppressed the growth of dual-drug resistant cells. **a** SKMEL28 DPR and WM2664 DPR were transduced with shRNA targeting nontarget (vector) or shRNA targeting against integrin α3, α11 and β1. Cell lysates were made for immunoblot analysis with antibodies indicated. β-ACTIN is as loading control. **b** Survival curves of SKMEL28 DPR and WM2664 DPR titrated with dasatinib for 72 h. Results are shown relative to DMSO-treated controls (mean ± SEM, *n* = 5; dashed line, 50% inhibition). **c** Long-term colony assays of resistant sub-lines treated with dasatinib as indicated concentration. **d** Quantification of (**c**) by Image J, ∗*p <* 0.05, ∗∗*p <* 0.01, or ∗∗∗*p <* 0.001. **e** Clonogenic assays of DPRs engineered control (vector) or Src-targeting shRNA treated with 1 μM AZD6244 and 0.1 μM BEZ235. **f** Survival curves of resistant sublines of SKMEL28 and WM2664 titrated with AZD6244 + BEZ235 with or without PF-562271 for 72 h. Results are shown relative to DMSO-treated controls (mean ± SEM, n = 5; dashed line, 50% inhibition). **g** Long-term colony assays of SKMEL28 DPR, WM2664 TPR and WM2664 DPR treated with PF-562271. **h** Quantification of (**g**) by Image J, ∗*p <* 0.05, ∗∗*p <* 0.01, or ∗∗∗*p <* 0.001

To unravel dual-drug resistant mechanisms mediated by integrins, we detected the activation of Src, one of the major downstream kinase of integrins [33–35]. Notably, RNA interference of integrins α3, α11, and β1 attenuated Src phosphorylation (Fig. 5a). We then investigated whether or not Src mediated MAPKi and PI3K/mTORi dual resistance. Short-term MTT assay (Fig. 5b) indicated that dasatinib, a selective Src family kinase inhibitor, sensitized SKMEL28 DPR, WM2664 TPR and DMR9 to AZD6244 plus BEZ235 by about 10-fold. Consistent with above results, long-term clonogenic assay showed that Src inhibitior suppressed dual-drug resistant sublines proliferation (Fig. 5c, d). However, their parental cell lines, SKMEL28 and WM2664, were not sensitive to dasatinib (IC50 around 10uM) (Supplementary Fig. 5a), suggesting that integrin-Src axis might not be a predominant pathway in parental cells. Considering that dasatinib can also inhibit other members in Src family such as Fyn and Lyn, we constructed shRNA targeted Src. In line with the results of small molecule inhibitor assay, knockdown of Src also sensitized the resistant sublines to the dual drugs treatment compared to control cells (Fig. 5e and Supplementary Fig. 5b-d).

We then detected the activation of focal adhesion kinase (FAK), another major downstream kinase of integrins [33, 34]. Western blot results revealed that integrin α3, α11, and β1 knockdown attenuated FAK phosphorylation in SKMEL28 DPR and WM2664 DPR (Fig. 5a). However, MTT assay (Fig. 5f and Supplementary Fig. 5e, f) and clonogenic assay (Fig. 5g, h and Supplementary Fig. 5g) with PF-562271 or Defactinib, FAK specific inhibitors showed that FAK inhibition failed to re-sensitize DPRs to MAPKi and PI3K/mTORi treatment, implying that the drug resistance may not rendered by FAK. Previous studies also proved that integrins regulated drug resistance and tumor progress through TGFβ, WNT, and NF-κB pathways [36–38]. However, both MTT assay and clonogenic assay showed that blockades of these pathways were not able to restore the sensitivity of SKMEL28 DPR and WM2664 DPR to MAPKi and PI3K/mTORi (Supplementary Fig. 5h-j). In conclusion, these results indicated that Src signaling pathway, rather than FAK, TGFβ, WNT, or NF-κB, rendered MAPKi and PI3K/mTORi dual-inhibitors resistance in melanoma.

### YAP1 pathway mediated integrin-Src axis signaling in MAPK/PI3K dual-inhibitor resistant melanoma cells

To explore the downstream pathways of Src in the resistant sublines, we probed the activations of potential Src-downstream proteins, including p-STAT3, p-c-JUN, p-AKT and p-p38 [39]. The results showed that p-p38, p-c-JUN, and p-AKT but not p-STAT3 were suppressed by Src inhibitor up to 24 h in SKMEL28 DPR (Supplementary Fig. 6b), suggesting that RhoA-p38-c-JUN pathway may mediated Src signaling [40]. Then, we tested whether or not blocking this pathway with the inhibitors can flip the MAPK and PI3K/mTOR dual resistance. To our surprise, the results showed that neither RhoAi nor p38i sensitized the resistant sublines to dual-drug treatment, suggesting that RhoA-p38-c-JUN pathway might not be functionally involved in the dual-resistance to MAPK and PI3K/mTOR inhibitors (Supplementary Fig. 6c, d). Moreover, STAT3i cannot inhibit resistant sublines growth in the short- and long-term proliferation assay either (Supplementary Fig. 6c, d).

**Fig. 6:**
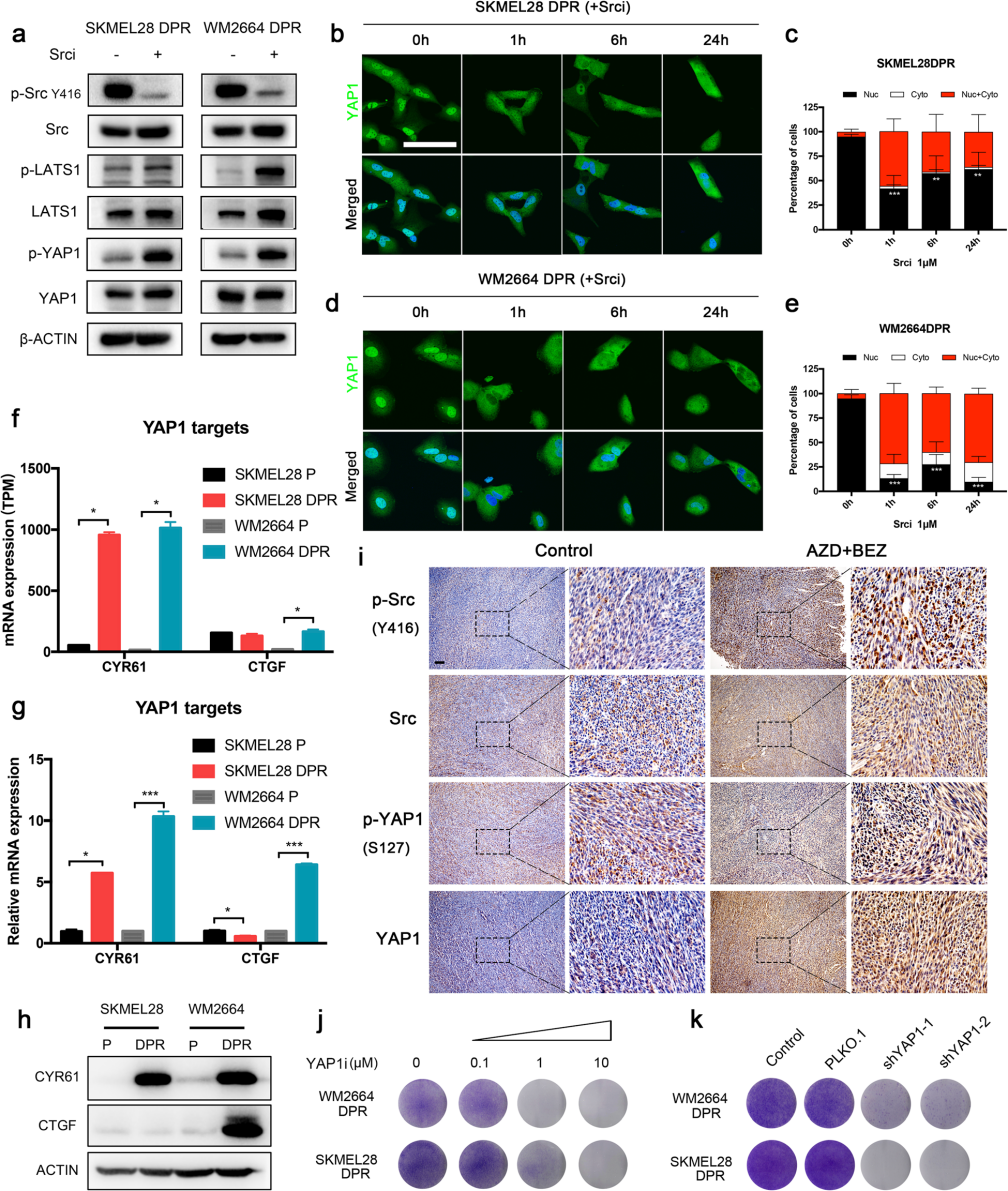
Src-YAP1 mediated the resistance to MAPK and PI3K/mTOR dual inhibitors. **a** Western blots showing indicated proteins levels in SKMEL28 DPR and WM2664 DPR treated with or without dasatinib (1 μM) for 1 h. **b-e** Immunofluorescence micrographs of YAP1 localization in SKMEL28 DPR (**b**) and WM2664 DPR (**d**) treated with dasatinib for indicated durations, scare bar = 100 μm. **c**, quantification of **b**, ∗*p <* 0.05, ∗∗*p <* 0.01, or ∗∗∗*p <* 0.001. **e**, quantification of **d**, ∗*p <* 0.05, ∗∗*p <* 0.01, or ∗∗∗*p <* 0.001. **f** TPM values of YAP1 targets (CTGF and CYR61) in SKMEL28 and WM2664 isogenic pairs, ∗*p <* 0.05. **g** mRNA expression of YAP1 targets (CTGF and CYR61) measured by q-PCR in SKMEL28 and WM2664 parental and DPRs, ∗*p <* 0.05, ∗∗*p <* 0.01, or ∗∗∗*p <* 0.001. **h** Western blots showing YAP1 targets protein levels in SKMEL28 and WM2664 parental and dual resistant sublines. **i** Representative images of IHC analysis with indicated proteins in tumors grown with or without AZD6244 plus BEZ235, scale bar = 100 μm. Image is representative of five independent experiments. **j** Clonogenic assays of DPRs were performed 10–14 days after treated with YAP1 inhibitor as indicated concentration. **k** Clonogenic assays of DPRs engineered control (vector) or YAP1-targeting shRNA treated with 1 μM AZD6244 and 0.1 μM BEZ235

Recently, it was reported that Src kinase mediated the ECM activated hippo pathway by directly phosphorylating LATS1 and YAP1 [41]. In line with this conclusion, we observed that dasatinib treatment induced phosphorylation of LATS1 and YAP1 in resistant sublines, which in turn lead to YAP1 cytoplasmic retention and subsequent degradation (Fig. 6a). YAP1 staining showed that nuclear enrichment of YAP1 was suppressed significantly in the presence of dasatinib. (Fig. 6b-e). Accordingly, the canonical YAP1 target gene CYR61 and CTGF were upregulated significantly in dual resistant sublines compared with their parental cells (Fig. 6f-h). In addition, we found that dual inhibitors treatment activated Src kinase in our xenograft melanoma model, which accompanied with the upregulation of YAP1 protein level (Supplementary Fig. 1d and Fig. 6i). Functional assay showed that YAP1 inhibitor significantly sensitized SKMEL28 DPR and WM2664 DPR to MAPK and PI3K/mTOR dual inhibitors in a dose dependent manner (Fig. 6j). Consistently, shRNA mediated YAP1 knockdown also suppressed the proliferation of dual-inhibitor resistant sublines (Fig. 6k and Supplementary Fig. 6a). Taken together, these results indicated that integrin/Src/YAP1 axis mediated resistance to MAPK and PI3K/mTOR dual inhibitors, which made it a promising target for the development of combinatorial regimens overcoming MAPK and PI3K/mTOR dual inhibitors–refractory melanoma patients.

## Discussion

Although multiple regimens, including immune therapy and chemotherapy, were combined with targeted therapy for overcoming the drug tolerant, blockade of genes driving resistance remains the most efficient way. PI3K/mTOR pathway is regarded as the predominant alternative signaling during the MAPK pathway inhibition. Increasing evidence has demonstrated that combination MAPK and PI3K/mTOR targeted therapy achieved promising antitumor effects in vitro and in vivo [24, 25, 42]. Multiple combinatorial regimens were designed and practiced including MEKi and PI3K/mTORi, MEKi and PI3Ki, PI3Ki and BRAFi et al. in clinics (https://clinicaltrials.gov). In line with these reports, we also observed high efficacy of this combination in vitro and in vivo. However, acquired resistance is also an inevitable impediment for combination therapy to produce durable clinical benefits. Therefore, new therapeutic strategies may need to be proposed that three or more complex drugs combination to target pathway independent of MAPK and PI3K/mTOR. In this study, we performed differential transcriptome analysis between parental and dual-resistant models and identified that activation of integrin pathway as a critical component to confer resistance to dual targeted MAPK and PI3K/mTOR pathways therapy for the first time.

**Fig. 7:**
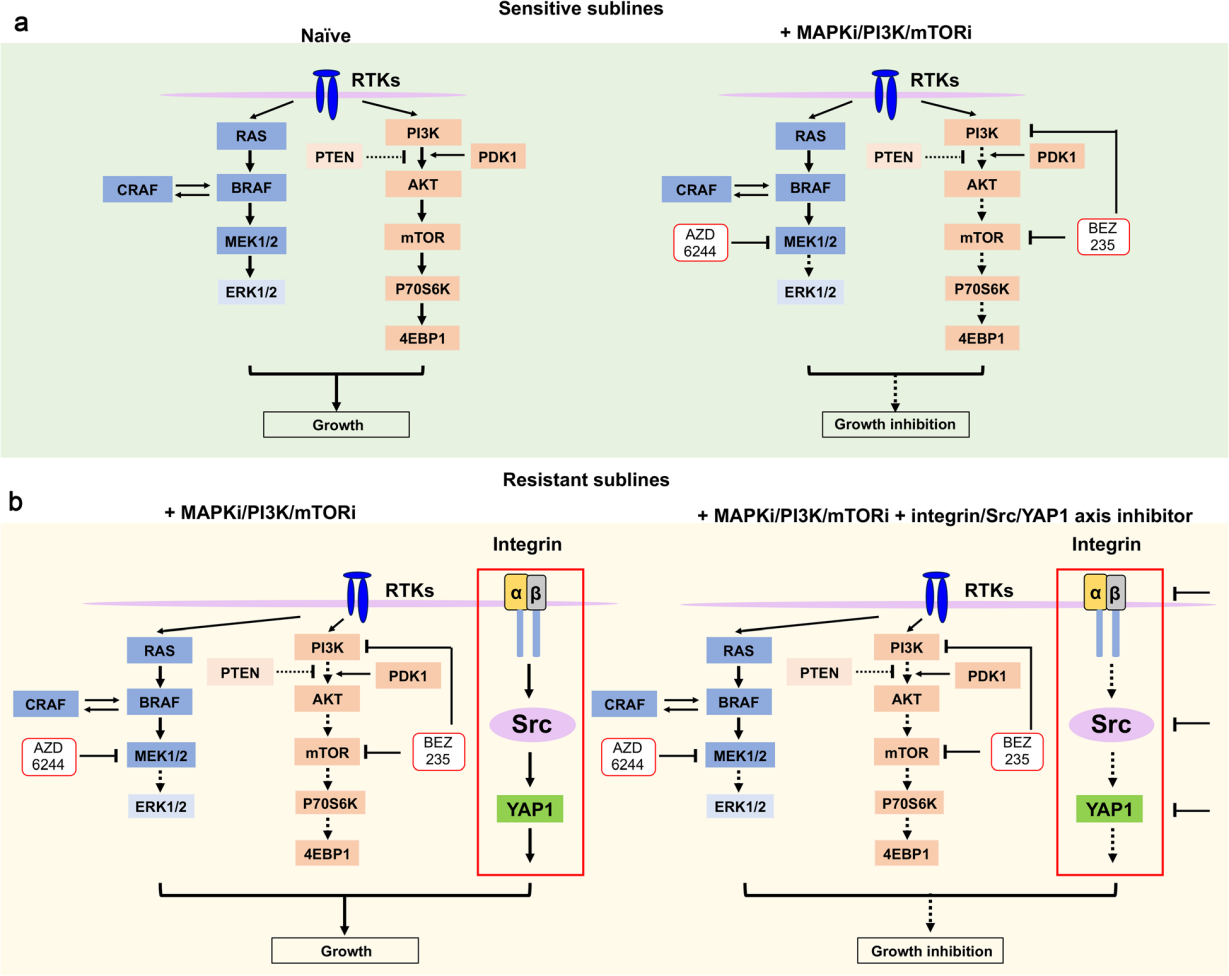
A proposed model depicting the role of integrin/Src/YAP1 axis in MAPK and PI3K/mTOR dual-inhibitor resistance. **a** In the sensitive cell lines, the MAPK or PI3K/mTOR pathway is continuously activated due to aberrant mutation or amplification, which leads to abnormal growth of melanoma cell. The combination of MAPK and PI3K/mTOR targeted therapy effectively induced melanoma cell death and inhibited the tumor growth. **b** In the MAPKi and PI3K/mTORi dual-resistant cell lines, the activation of integrin/Src/YAP1 signaling renders tolerance to MAPK and PI3K/mTOR dual inhibitors and inhibition of integrin or Src or YAP1 is capable to restore melanoma cell sensitivity to MAPKi and PI3K/mTORi, which leads to tumor growth inhibition

Integrin family is one of the major adhesion receptors [43] and consist of 24 heterodimeric αβ receptors, which regulates interaction between cell and extracellular matrix or between cells by counter-receptors on cell surface [34]. Upregulation of integrins not only associated with tumor progression and metastasis, but also could strengthen the ability of cancer cells to escape targeted therapy, involving in the resistance to bevacizumab [44], lapatinib [45], and erlotinib [46]. Our data showed that integrins α3, α11, and β1 were upregulated both in dual resistant models in vitro and in vivo. Additionally, knockdown of these integrins restored sensitivity to MEKi and PI3K/mTORi in resistant sublines. Integrin β1 was reported to promote resistance to RTKs targeted therapies through maintaining ERK and PI3K/AKT pathways [45, 47]. However, AZD6244 and BEZ235 continued to block p-ERK, p-S6, and p-4E-BP1 in dual resistant models (Fig. 2a and Supplementary Fig. 2a), implying that integrin confers resistance through alternative redundant pathway independent of canonical PI3K or MAPK cascades. Recent research showed that ECM/Integrin β1/Src/FAK signaling play a vital role in the resistance to HER2 and PI3K inhibitors [31]. Similar to these results, we observed that the expression of collagens, fibronectin, vitronectin, and laminin were increased in DPRs (Fig. 3e). However, the role of ECM components in resistance to MEKi and PI3K/mTORi in our models should be further illustrated, though the RGD peptide, a potential inhibitor of integrins with its motif Arg-Gly-Asp [48], failed to inhibit the growth of SKMEL28 DPR (data not show). Besides, the knockdown of integrin would suppress the ability of melanoma cells to bind a spectrum of ECM ligands [44]. Functional investigation identified that ECM stimulated integrin α3β1 and α11β1 further activated Src and YAP1 signaling, which compensate the inhibition of MAPK and PI3K/mTOR pathways. Co-targeting MAPK/PI3K pathway with either integrins, or Src, or YAP1 synergistically inhibited proliferation of melanoma cell lines (Fig. 7).

Integrin/FAK/Src signaling was reported as a potential mechanism for BRAFi single-drug resistance [49]. In our study, the MAPKi/PI3Ki dual-resistance mechanism was proved to be driven by integrin/Src axis independent of FAK. A potential explanation is that PI3K inhibitor blocks the downstream signaling of FAK and change the dependency of cellular signaling in dual-resistant melanoma cells. Alternatively, YAP1 pathway was identified in our model as the downstream of integrin/Src axis. Therefore, our study provided two alternative targets for therapy of melanoma which tolerated primary oncogene targeted therapy.

PI3K/AKT/mTOR pathway is regarded as predominant compensatory signaling when primary driver pathway MAPK is blocked. In this study, we sought for the alternative pathway in addition to them. Our conclusion not only provided the molecular insight to mechanism for drug resistance, but also proposed a potential resolution for the current clinical trials setback. Recently, several clinical trials of MEKi plus PI3K/mTORi in subjects with advanced solid tumors were failed due to the serious adverse events, although the regimens showed promising activity [50, 51]. The potential reason is that the ranges of dose between efficacy and toxicity are too small which results in the therapeutic indexes of MEKi and PI3K/mTORi are unacceptable. Efforts on either increase the specificity or increase the efficacy will improve the clinical performance. In our study, we found that inhibition of YAP1 or Src sensitized the melanoma cells to MEKi plus PI3K/mTORi. Although knockdown of Src with shRNA inhibited the proliferation, further research needs to demonstrate whether other members in Src family play a role in resistance to dual pathway inhibition as dasatinib is a pan inhibitor which can suppress Src and BCR/ABL family kinases. These results suggested that combinatorial regimens with MEKi plus PI3K/mTORi plus Srci (or YAP1i) would limit adverse effect by diminishing drug doses.

## Materials and methods

### Cell culture

SKMEL28, WM2664, SMM102, and HEK293T were cultured in DMEM medium (Hyclone) supplemented with 10% fetal bovine serum (FBS) and 1% penicillin/streptomycin (100 μg/mL, Hyclone) in a humidified 5% CO_2_ incubator at 37°C. WM2664 DMRs were generated by treating WM2664 with 1 μM AZD6244 and 0.1 μM BEZ235 for about 6 months. Serval surviving colonies treated with AZD6244 and BEZ235 were selected using cloning rings and named DMR#1, #2 etc. WM2664 DPR, SKMEL28 DPR were generated by treating parental cells with increasing concentration of AZD6244 and BEZ235. Cells with the ability to grow in 1 μM AZD6244 and 0.1 μM BEZ235 were obtained about 8 months after initial drugs exposure. Dual-drug resistant cell lines were maintained in the continuous presence of 1 μM AZD6244 and 0.1 μM BEZ235.

### Reagents and antibody

AZD6244 (MEKi), BEZ235 (PI3K/mTORi), CCG-1423 (RhoAi), QNZ (EVP4593) (NF-κBi), C188–9 (STAT3i), dasatinib (Srci), verteporfin (YAP1i), XAV-939 (WNTi), SB525334 (TGFβi), doramapimod (p38i), Defactinib (FAKi), and PF-562271 (FAKi) were purchased from SELLECK. Stocks and dilutions of drugs were made in dimethyl sulfoxide (DMSO, sigma). Antibodies against phospho-MAPK (ERK1/2) (Thr202/204), MAPK (ERK1/2), MEK1/2, p-MEK1/2 (Ser217/221), p-c-JUN (Ser73), c-JUN, p-p38 (Thr180/Tyr182), p38, p-Src (Y416), Src, p-YAP1 (Ser127), YAP1, p-LATS1 (Ser909), LATS1, AKT, p-AKT (Thr308/Ser473), p-P70S6K (Thr389), P70S6K, S6, p-S6 (Ser240/244), p-STAT3 (Y727), STAT3, p-FAK (Tyr397), FAK, p-mTOR (Ser2448), m-TOR, p-4E-BP1 (Thr37/46), and 4E-BP1 were purchased from Cell Signaling Technology (CST); antibodies against tubulin and β-ACTIN were purchased from Santa Cruz and ZsBio.

### Immunoblot analysis

For western blot, cells were washed with ice-cold PBS twice and lysed in RIPA buffer (#20–188, Millipore) with phosphatase inhibitor and protease inhibitor (cocktail). Lysate were quantified (Bicinchoninic Acid assay, Sigma), normalized, denatured (98°C) and resolved by SDS gel electrophoresis on 8–10% Tris-Glycine gels. Then protein was transferred to PVDF membranes (Millipore) and probed with primary antibodies. Secondary antibodies (goat anti-rabbit and anti-mouse) are HRP-linked. Western lightning ECL reagent (Millipore) was used for signal detection. Chemiluminescence signal was acquired by ChemiDoc MP imaging systems (Bio-rad) which use charge-coupled device (CDD) cameras to capture the luminescent signals on western blots.

### Cell cycle and apoptosis

For cell cycle analysis, cells were harvested and washed with ice-cold PBS, then fixed by 70% ice-cold ethanol over night at 4°C, nuclear stained with propidium iodide (PI). Cell suspension was immediately analyzed by flow cytometer. Data analysis was analyzed by ModFit software. For cell apoptosis analysis, cells were stained with Annexin V-FITC (10 min) and propidium iodide (5 min) (4A Biotech) at room temperature in the dark. Data analysis was analyzed by CytExpert 2.0 software.

### Cell viability assay

Cells were seeded in 96-well plates at 2000–3000 cells/well and drugs treated on the following day. Cells were then incubated for another 72 h and cell viability was measured using MTS (CellTiter 96 AQueous One Solution Cell Proliferation Assay, Promega) following the manufacturer’s recommendations. Relative survival in the presence of indicated drugs were normalized to DMSO after background subtraction. Survival curve was performed by GraphPad Prism 7.0 software.

### Clonogenic assay

Cells were seeded into 6-well plates (1-2 × 10^4^ Cells per well) and allowed to adhere overnight. On the following day, cells were treated with several inhibitors. Media and drugs were replenished every 2 days. Colonies were fixed in 4% paraformaldehyde and stained with 0.05% crystal violet after 10–14 days. Quantitative analysis was performed by Image J software.

### Immunofluorescence

Preparing the slides into the 24-well plates and cell were seeded on the slides overnight, cells fixed with 4% paraformaldehyde for 40 min at room temperature after inhibitors treatment. After fixation, 0.2% Triton X-100 was applied for permeabilization. The permeabilized cells were blocked in blocking buffer (1% BSA) for 60 min at room temperature. After that, cells were incubated with primary antibodies overnight at 4°C. Bound primary antibodies were detected by incubating with Alexa Fluor 488-conjugated secondary antibodies (Invitrogen Cat#A-21206) for 1 h at room temperature. For staining WGA and phalloidin, the fixed cells were stained with 5 μg/mL WGA Alexa Fluor® 488 conjugate for 10 min or 0.1 μg/mL phalloidin-FITC for 30 min at room temperature. Fluorescence images were acquired using inverted fluorescence microscope (ZEISS) and confocal microscopy (Nikon). Quantification of YAP1 was evaluated as described previously [52].

### Lentiviral shRNA constructs virus infection

Lentivirus preparations were produced by co-transfecting helper virus packaging plasmids pMD. G, RSV-REV, pMDLg/p and pLKO.1 puro (empty vector or containing shRNA) into 293 T cells. Cells were seeded into 6-well or 12-well plates and infections were carried out in the presence of 2 μg/mL protamine. Following transduction, WM2664 DPR and SKEML28 DPR were treated with 2 μg/mL puromycin for selecting stable expression of shRNA or control vector.

### RNA isolation, RNA sequencing and analysis

Total RNAs were isolated using TRIZOL (Invitrogen) from cells (parental and resistant cell lines) following the manufacturer’s recommendations. RNA seq libraries were generated with TruSeq RNA Library Prep Kit according to the manufacture’ protocol. Enriched RNA-seq libraries were multiplexed and sequenced on the Illumina HiSeq 4000 platform. Sequencing reads were aligned to human reference genome GRCh38(ftp://ftp.ensembl.org) and quantified by Salmon v0.81. Raw read counts of genes were used as input for DESeq2 v1.24.0 to identify differentially expressed genes (DEGs), differential gene expression analysis was performed using a generalized linear model with the Wald statistical test. DEGs were defined with *p* < 0.05 & | log2 (fold change) | > 1. Then DEGs were used to conducted Gene Ontology (GO) and Kyoto Encyclopedia of Genes and Genomes (KEGG) enrichment analysis with ClusterProfiler v3.1.2.0 R package with default parameters. Top 5 pathways with the smallest *p*-value were selected to plot as chord diagram using Circlize v0.4.8 R package. The hypergeometric test method is Fisher’s exact test, and the FDR correction method is Benjamini-Hochberg.

### Principal component analysis

Gene expression (TPM) data was used as input of Principal Component Analysis (PCA). Genes that expressed in > 50% groups (expressed genes) were selected, the TPM matrix was transformed to log2(TPM + 1) and then scaled. PCA was performed using the prcomp function in R.

### Protein-protein interaction networks functional enrichment analysis

To find kernel genes in ECM pathway regulation, we selected significantly changed genes in the ECM pathway of SKMEL28 or WM2664. Then protein-protein interaction networks functional enrichment result was obtained from STRING, and the result was visualized using the Cytoscape v3.7.1 software, genes with more than 1 degree and edges with confidence > 0.9 were kept.

### Quantitative real-time PCR

Total RNA was extracted from cells using TRIZOL (Invitrogen). cDNA was synthesized with the Revert Aid First Strand cDNA Synthesis Kit (Thermo Fisher Scientific) using 1 μg of total RNA as a template. Quantitative PCR was performed using Universal SYBR® Green Super mix (Bio-Rad). ACTIN was used as reference gene for relative quantification. Primers used for qPCR are listed in Supplemental Table 2.

### Immunohistochemistry (IHC) staining

To prepare the tumor samples for IHC staining, the tumor pieces were fixed with 10% formalin followed by paraffin embedding. Tumor sections of 4 μm thickness were mounted on glass slides for IHC staining as described previously [28]. For p-S6, p-4E-BP1, 4E-BP1, p-ERK, integrin α3, integrin α11, integrin β1, p-Src, Src, p-YAP1, YAP1 immunohistochemistry staining, the slides were deparaffinized, incubated in 3% hydrogen peroxide, antigen retrieval was performed in EDTA (pH = 9.0) or citrate (pH = 6.0) for 3 min in pressure cooker. The slides incubated with primary antibodies of interest overnight, followed incubation with appropriate HRP-conjugated secondary antibodies at room temperature for 30 min. At last, the slides were incubated with DAB (3,3′-diaminobenzidine) for visualization.

### In vivo mouse studies

SMM102 cell line derived from transgenic Braf^V600E^/wt, Cdkn2−/−, Pten−/− mouse as described [28]. Female C57BL/6 mice were obtained from Beijing HFK Bioscience Co.,Ltd. (Beijing, China). The animals were housed and maintained under specific pathogen-free conditions in facilities and treated humanely throughout the studies. All animal experiments were performed according to the protocols approved by the Ethics Review Committee of Animal Experimentation of Sichuan University. 5–7 weeks old mice were injected with 1 × 10^5^ SMM102 cells. Two sites on the flanks were injected per mouse. Tumor volumes were measured in two dimensions (length and width) with calipers every two days. Tumor sizes were calculated by the standard formula of tumor size = (length × width^2^) / 2. Body weights and tumor weights were measured by the balance. Mice that developed tumors reaching 150–200 mm^3^ in size were randomized into two groups with five mice in each group: vehicle, 25 mg/kg AZD6244 plus 5 mg/kg BEZ235. AZD6244, solubilized in a methocel/polysorbate buffer, was injected once every two days by intraperitoneal injection at the dose of 25 mg/kg with BEZ235 together. BEZ235, was reconstituted in NMP (1-methyl-2 pyrrolidone) and PEG300, and injected once every two days by intraperitoneal injection at the dose of 5 mg/kg with AZD6244 together.

### Statistical analysis

Data are presented as mean ± SD unless otherwise stated. Significance was determined with GraphPad Prism 7 software using the Student’s t-test or ANOVA where ∗*p <* 0.05, ∗∗*p <* 0.01 or ∗∗∗*p <* 0.001.

## Supplementary information

**Additional file 1: Supplementary Table 1**. shRNA sequence. **Supplementary Table 2.** q-PCR primers. **Supplementary** **Figure 1.** Combined AZD6244 and BEZ235 suppressed the proliferation of melanoma cells *in vitro* and *in vivo.* **Supplementary** **Figure 2.** Combination AZD6244 and BEZ235 inhibited MAPK and PI3K/mTOR pathways. **Supplementary** **Figure 3.** Transcriptome profiling revealed the differential genes in DPR resistant cell lines. **Supplementary** **Figure 4.** The knockdown efficiency of shRNA targeting integrins. **Supplementary** **Figure 5.** Exploring downstream pathways of integrins in DPRs. **Supplementary Figure 6.** Exploring downstream pathways of Src in DPRs

## Data Availability

All data generated or analyzed during this study are included in this published article and its supplementary information files.

## Abbreviations

BRAF: Serine/threonine-protein kinase
CAMs: Cell adhesion molecules
CST: Cell Signaling Technology
DEGs: Differentially expressed genes
DMR: Dual-monoclonal resistance
DPR: Dual-polyclonal resistance
ECM: Extracellular matrix
FAK: Focal adhesion kinase
FBS: Fetal bovine serum
GO: Gene Ontology
IHC: Immunohistochemistry
KEGG: Kyoto Encyclopedia of Genes and Genomes
MAPK: Mitogen-activated protein kinase
MEK: Mitogen-activated protein kinase kinase
mTOR: The mammalian target of rapamycin
NMP: 1-methyl-2 pyrrolidone
PCA: Principal Component Analysis
PI: Propidium iodide
PI3K: Phosphatidylinositol-3-kinase
SDR: Single drug resistant sublines
TPR: Triple drugs resistant models
