# Supplementary material for: Integrin-Src-YAP1 signaling mediates the melanoma acquired resistance to MAPK and PI3K/mTOR dual targeted therapy
Source: Mol Biomed. 2020 Nov 10;1:12. doi: 10.1186/s43556-020-00013-0 (PMC8607431; doi:10.1186/s43556-020-00013-0)
Supplement: Supplementary file 1 — Additional file 1: Supplementary Table 1. shRNA sequence. Supplementary Table 2. q-PCR primers. Supplementary Figure 1. Combined AZD6244 and BEZ235 suppressed the proliferation of melanoma cells in vitro and in vivo. Supplementary Figure 2. Combination AZD6244 and BEZ235 inhibited MAPK and PI3K/mTOR pathways. Supplementary Figure 3. Transcriptome profiling revealed the differential genes in DPR resistant cell lines. Supplementary Figure 4. The knockdown efficiency of shRNA targeting integrins. Supplementary Figure 5. Exploring downstream pathways of integrins in DPRs. Supplementary Figure 6. Exploring downstream pathways of Src in DPRs [file 43556_2020_13_MOESM1_ESM.docx]

**Supplementary information**

Supplementary Table 1. shRNA sequence

| Target of shRNA | Sequence |
| --- | --- |
| Src-sh1 | CATCCTCAGGAACCAACAATT |
| Src-sh2 | GACAGACCTGTCCTTCAAGAA |
| ITGA3-sh1 | ACAGCAGAGACGTCCGGAAAT |
| ITGA3-sh2 | GACCTCGCTTAGCATGGTAAA |
| ITGA11-sh1 | GCTCTTACTTTGGGAGTGAAA |
| ITGA11-sh2 | GCACGACATCAGTGGCAATAA |
| ITGB1-sh1 | GCCTTGCATTACTGCTGATAT |
| ITGB1-sh2 | TTTGTAGGAAGAGGGATAATA |
| YAP1-sh1 | GCCACCAAGCTAGATAAAGAA |
| YAP1-sh2 | TGATTGGTGAACATTTAACTGGG |

Supplementary Table 2. q-PCR primers

| Target genes | Primers |
| --- | --- |
| ACTIN | F: CATGTACGTTGCTATCCAGGC |
|  | R: CTCCTTAATGTCACGCACGAT |
| ITGA3 | F: CACCTTCATCGAGGATTACA |
|  | R: AGTCAATGTCCACAGAGAAC |
| ITGA11 | F: ATGAAAATCATGGTCAACGC |
|  | R: CTACAATGATCCAGATGGGG |
| ITGB1 | F: CCTTCTATTGCTCACCTTGT |
|  | R: TACTGCTGACTTAGGGATCA |

**Supplementary figures**


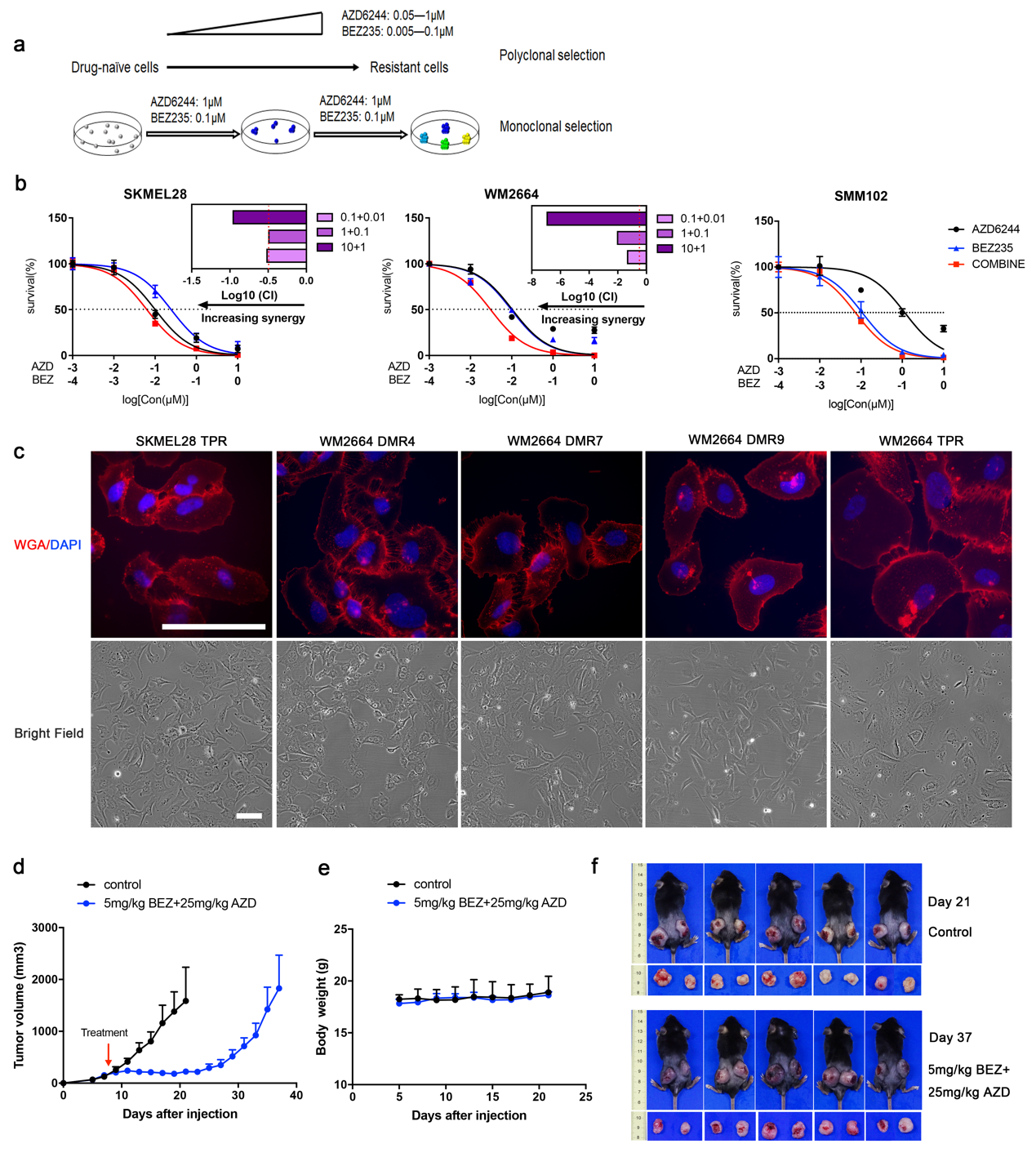


**Fig S1 Combined AZD6244 and BEZ235 suppressed the proliferation of melanoma cells *in vitro* and *in vivo***

**(a)** Drug naïve cells were chronically treated with increasing concentration of MEK inhibitor AZD6244 and PI3K/mTOR inhibitor BEZ235 or certain concentration of dual inhibitors. **(b)** Survival curves of SKMEL28, WM2664, and SMM102 titrated with AZD6244, BEZ235 or their combine, relative synergy of AZD6244 and BEZ235 combination in indicated concentrations. (mean±SEM, n = 5; dashed line, 50% inhibition). **(c)** Immunofluorescence staining for visualizing cell boundaries by fluorescence microscope (top), phase-contrast images showing morphological changes in WM2664 and SKMEL28 resistant sublines(down), (scale bar=100μm). **(d)** A total of 10^5^ cells were injected s.c. into the flanks of C57 mice (n=5), and animals were treated with control or 5mg/kg BEZ235 and 25mg/kg AZD6244 via i.p twice a day, starting 8 d after cells injection. Average tumor volumes are shown. **(e)** Body weight of tumor-bearing C57 mice (n=5). **(f)** Images of tumors on day 21 (control) and on day 37 (5mg/kg BEZ+25mg/kg AZD).


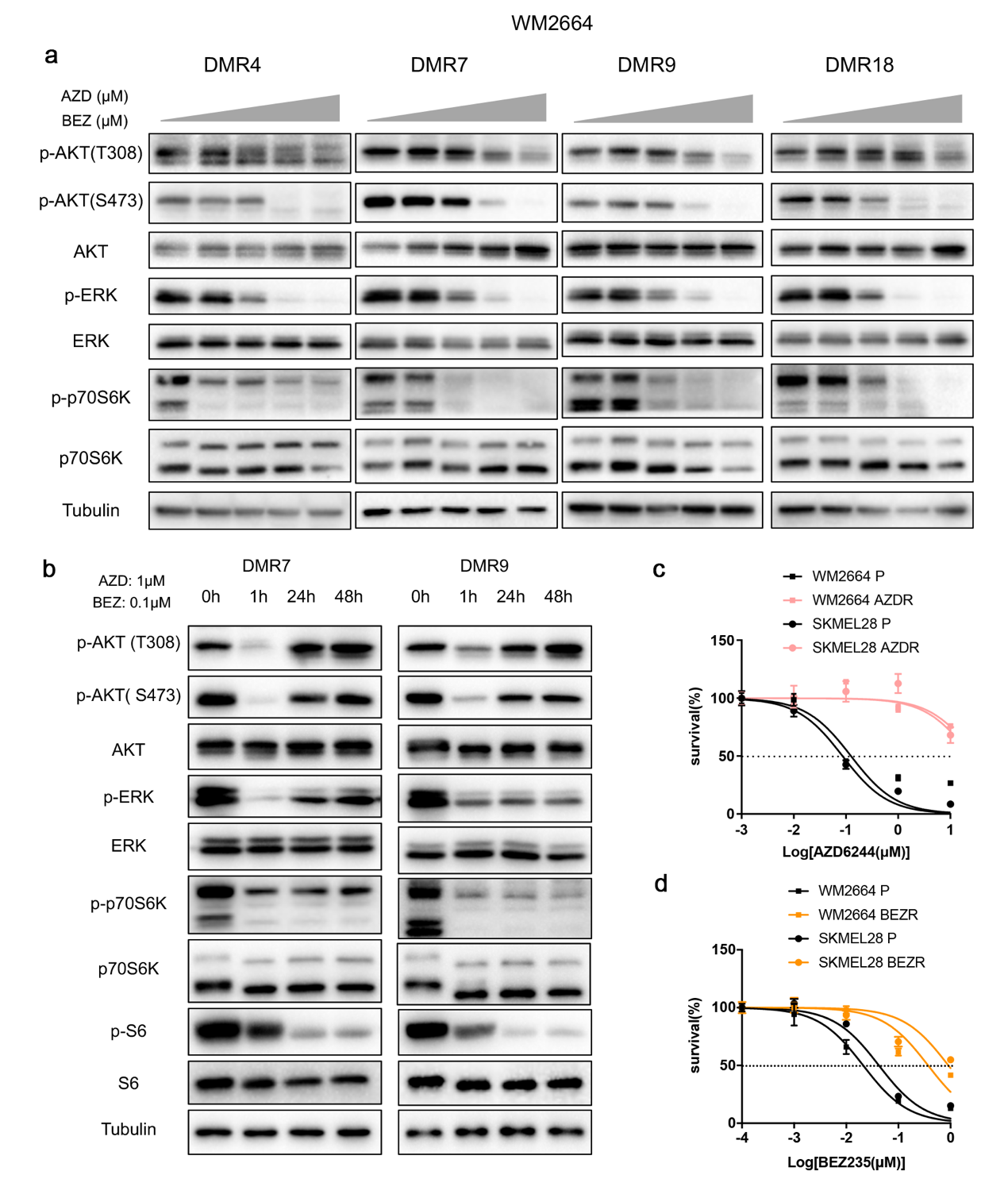


**Fig S2 Combination AZD6244 and BEZ235 inhibited MAPK and PI3K/mTOR pathways**

**(a)** WM2664 DMRs were treated with indicated concentrations of AZD6244 and BEZ235 for 1 hour. Cell lysates were analyzed by immunoblotting. **(b)** WM2664 DMRs were treated with AZD6244 (1μM) and BEZ235 (0.1μM) for indicated durations(h). Cell lysates were analyzed by immunoblotting. **(c)** Survival curves of parental and AZD6244 resistant cell lines (AZD-R) of SKMEL28 and WM2664 titrated with the AZD6244 for 72 hours. (mean±SEM, n = 5; dashed line, 50% inhibition). **(d)** Survival curves of parental and BEZ235 resistant cell lines (BEZ-R) of SKMEL28 and WM2664 titrated with the BEZ235 for 72 hours. Results are shown relative to DMSO-treated controls (mean±SEM, n = 5; dashed line, 50% inhibition)

**
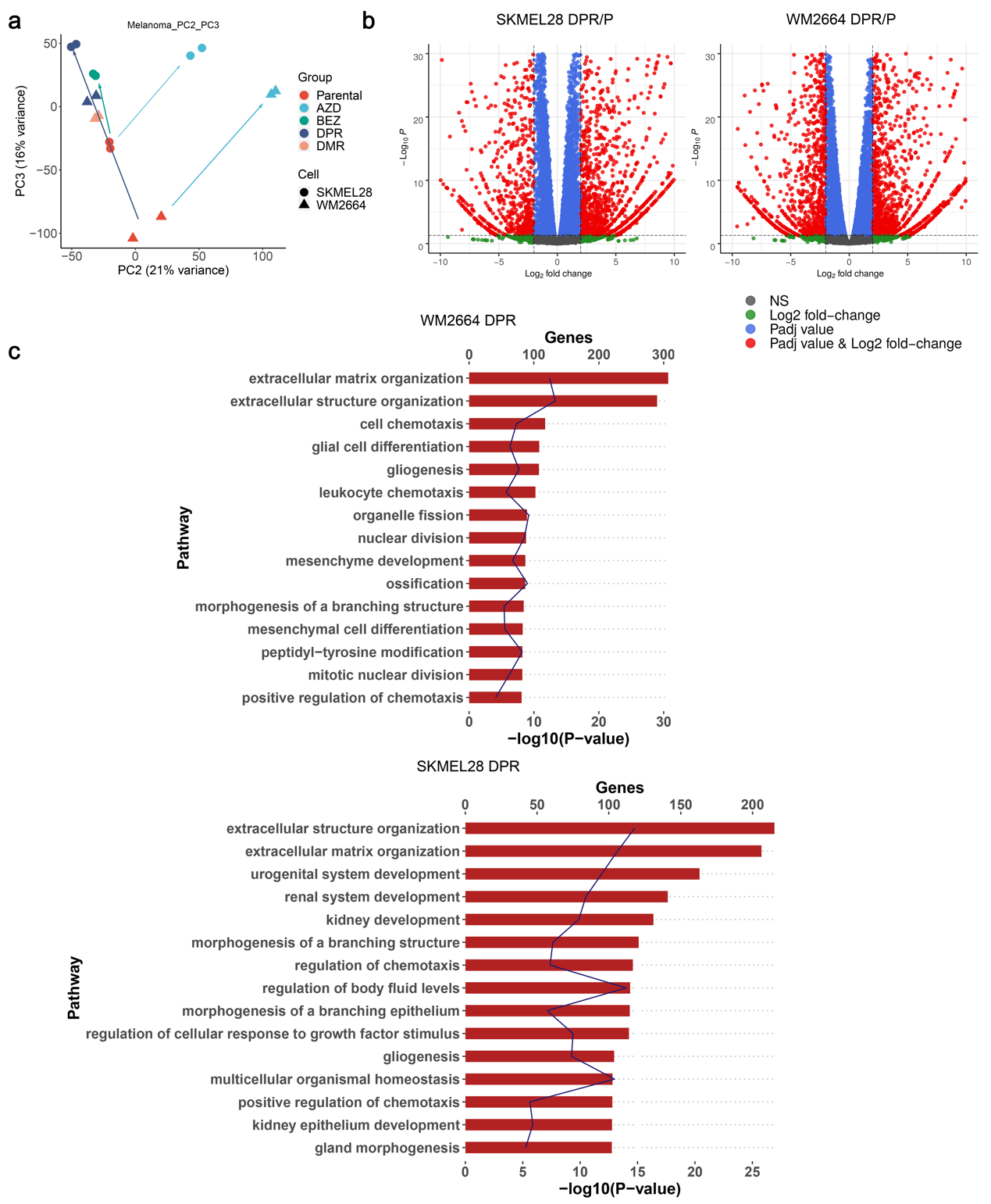
**

**Fig S3 Transcriptome profiling revealed the differential genes in DPR resistant cell lines**

**(a)** PCA analysis of RNA-seq profiles of WM2664 and SKMEL28 parental, DPRs, and single drug resistance (SDR). Each dot represents one sample. **(b)** Volcano plot of differential expressed genes between resistant and parental cells (*p* < 0.05, |Log2 (Fold change)| > 2 ). **(c)** GO analysis in differential expression genes between resistant and parental cells (*p* < 0.05, top 15).


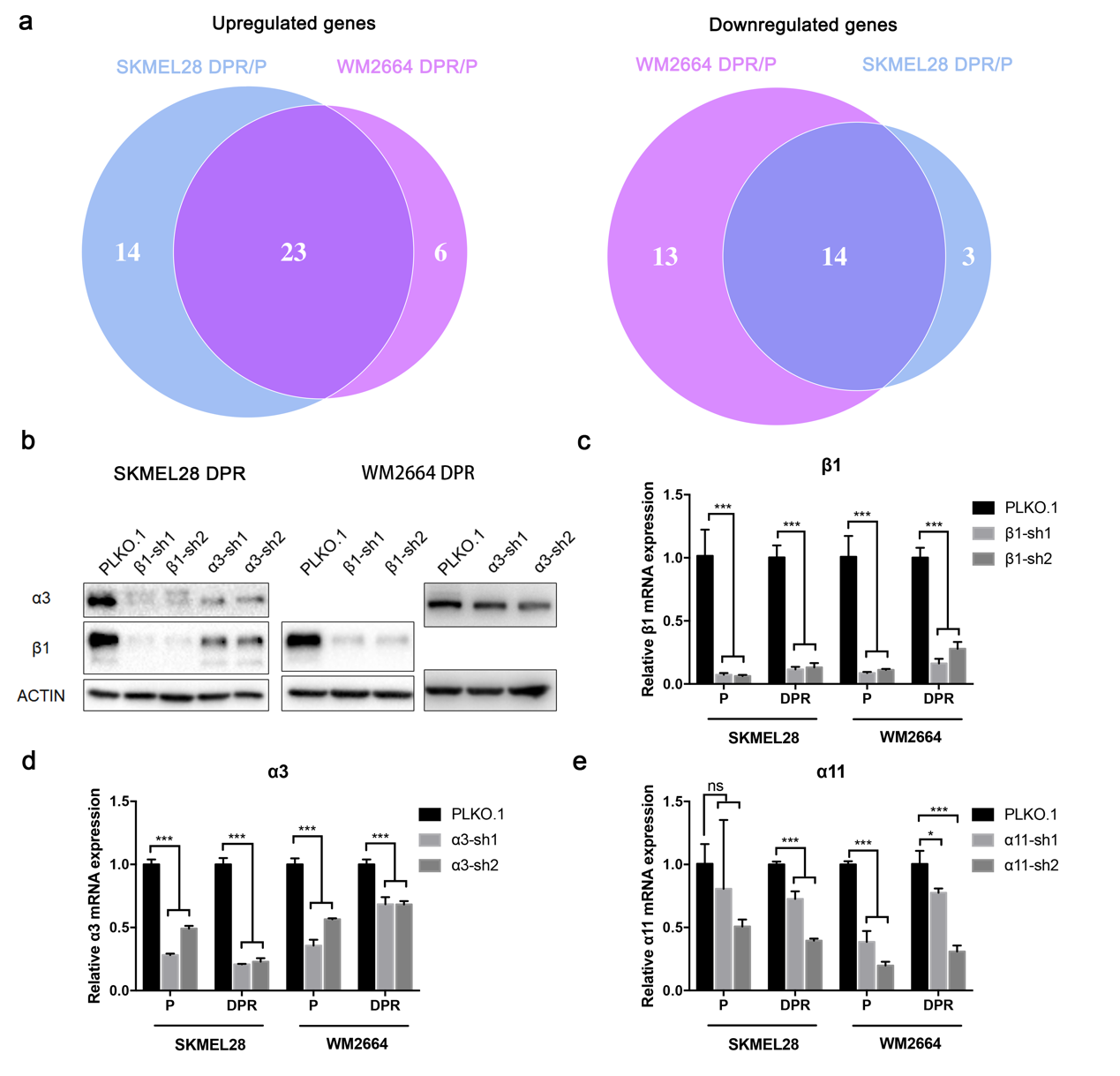


**Fig S4 The knockdown efficiency of shRNA targeting integrins**

**(a)** Venn diagram of differential expressed genes in ECM terms. **(b)** Western blot analysis of integrin α3 and β1 in SKMEL28 DPR and WM2664 DPR stably transfected with control or their individual shRNAs, β-ACTIN was as loading control. **(c-e)** mRNA expression of integrins β1, α3, and α11 in SKMEL28 and WM2664 parental and DPR sublines with shRNAs, respectively. Results are shown for one representative of three biological replicates. Significance was determined using One-way ANOVA, ∗*p* < 0.05, ∗∗*p* < 0.01, or ∗∗∗*p* < 0.001.


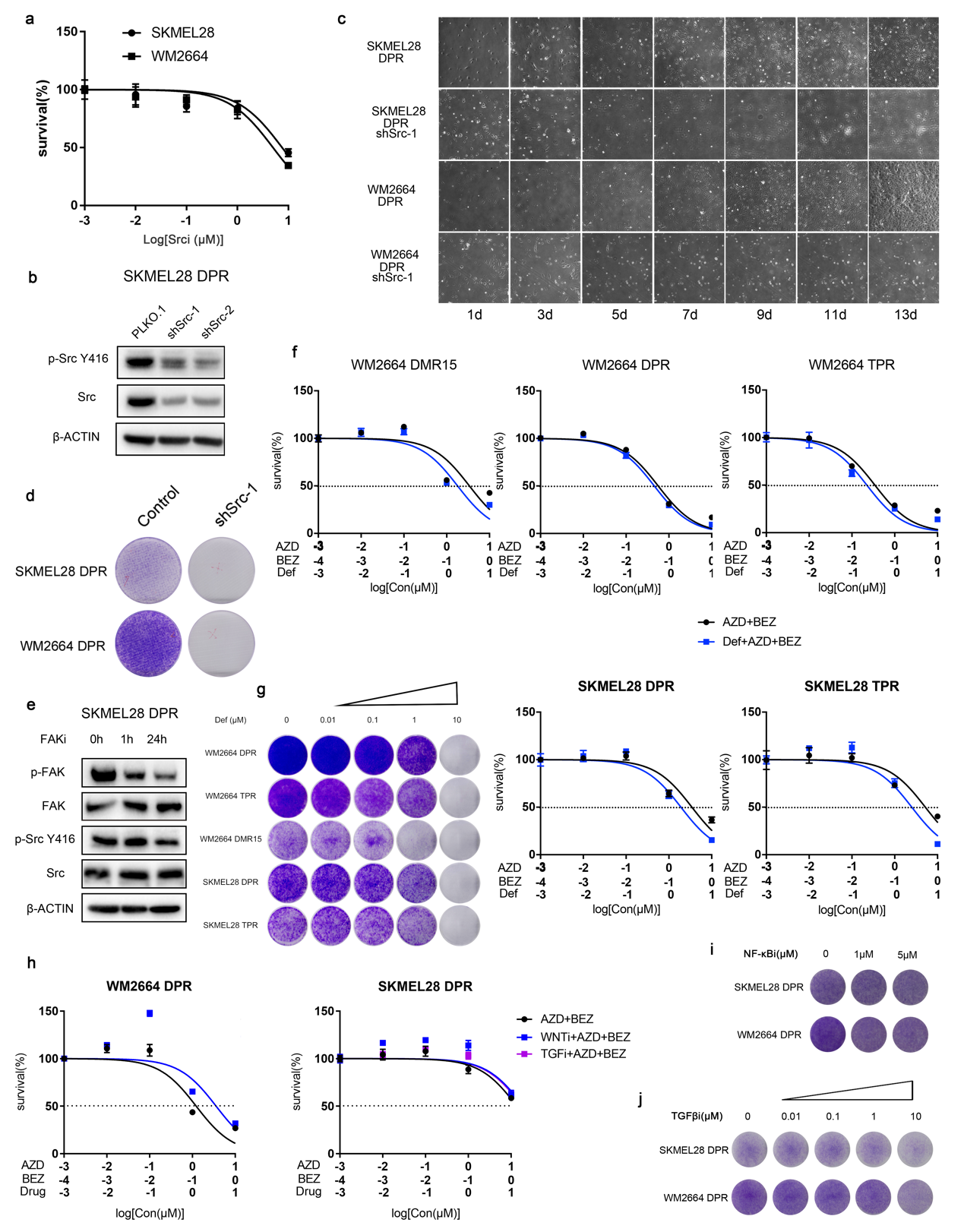


**Fig S5 Exploring downstream pathways of integrins in DPRs**

**(a)** Survival curves of SKMEL28 and WM2664 titrated with dasatinib for 72 hours. Results are shown relative to DMSO-treated controls (mean±SEM, n = 5; dashed line, 50% inhibition). **(b)** SKMEL28 DPR were transfected with vector (control) or shRNA targeting Src for 72h. The cell lysates were made for western blot analysis of indicated proteins. **(c)** Growth properties of DPRs engineered control (vector) or Src-targeting shRNA treated with 1μM AZD6244 and 0.1μM BEZ235. **(d)** Crystal violet stain of (c) in day 13. **(e)** SKMEL28 DPR was treated with PF-562271 (1μM) for the indicated time and phosphorylation of FAK and Src were assessed by immunoblot. **(f)** Survival curves of resistant sublines of SKMEL28 and WM2664 titrated with AZD6244 + BEZ235 with or without Defactinib for 72 hours. Results are shown relative to DMSO-treated controls (mean±SEM, n = 5; dashed line, 50% inhibition). **(g)** Long-term colony assays of resistant sublines treated with Defactinib. **(h)** Survival curves of DPRs titrated with AZD6244 + BEZ235 with or without WNT, TGFβ inhibitors for 72 hours. Results are shown relative to DMSO-treated controls (mean±SEM, n = 5; dashed line, 50% inhibition). **(i**-**j)** Long-term culture assay of SKMEL28 DPR and WM2664 DPR treated with NF-kB inhibitor (i) and TGFβ inhibitor (j) as indicated concentration.

**
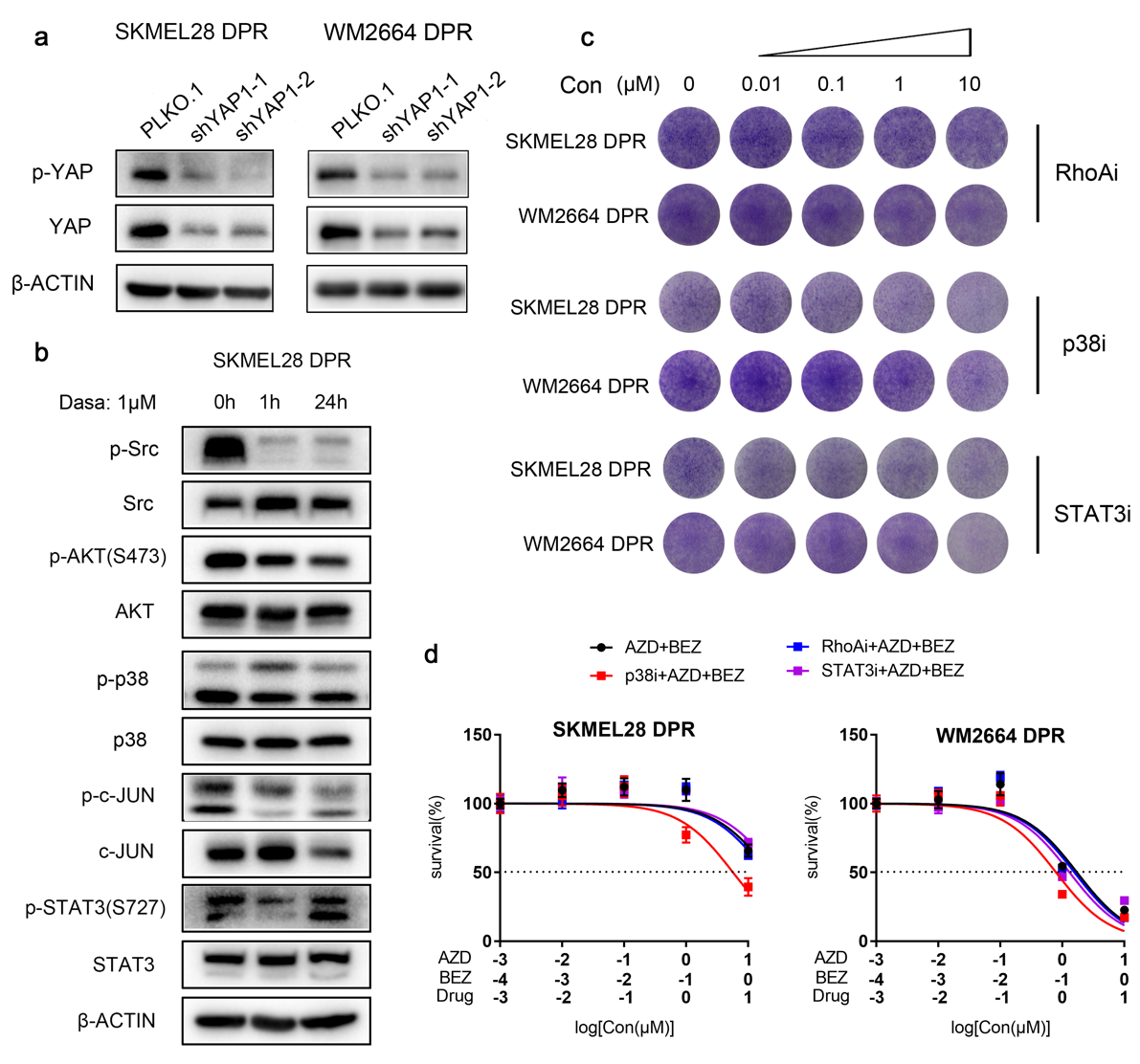
**

**Fig S6 Exploring downstream pathways of Src in DPRs**

**(a)** SKMEL28 DPR and WM2664 DPR were transfected with vector (control) or shRNA targeting YAP1 for 72h. The cell lysates were made for immunoblot analysis of indicated proteins. **(b)** Western blot showing indicated proteins levels in SKMEL28 DPR treated with dasatinib (1μM) for indicated durations (h). **(c)** Colony growth assays of SKMEL28 DPR and WM2664 DPR were performed 10-14 days treated with RhoA, p38, and STAT3 inhibitors as indicated concentration. **(d)** Survival curves of SKMEL28 DPR and WM2664 DPR titrated with AZD6244 + BEZ235 with or without RhoA, p38, STAT3 inhibitors for 72 hours. Results are shown relative to DMSO-treated controls (mean± SEM, n = 5; dashed line, 50% inhibition).
